# Dr. Jenner, on the Effects of Cutaneous Eruptions

**Published:** 1804-08-01

**Authors:** Edward Jenner

**Affiliations:** Berkeley


Medical and Phyfical Journal.

VOL. XII.] AUGUST 1, 1804. [NO. LXVI.

*Printed for R. PHILLIPS, by W. Thorne, Red Lion Court, Fleet Street, London.*

### *To the Editors of the Medical and Phyfical Journal.*

GENTLEMEN,

THOSE herpetic affections which so frequently appear among the children of the poor, and which are evidently contagious, often prevent the vaccine virus from producing its correct action. The skin, although it be apparently sound at the point of insertion, is nevertheless so influenced by the disease, as frequently to baffle all our efforts to produce a correct pustule, and consequently to secure the constitution from the contagion of the small-pox. The eruptions I allude to, for the most part, correspond with those of the Second Order of Cutaneous Diseases described by the ingenious Dr. WILLAN, under the term *Psoriasis Diffusa.** The face, the eye-lids, the tender skin behind the ears, and particularly the scalp, are the parts most commonly affected; but the limbs and body not unfrequently exhibit the same appearances. As far as I have been able to observe, it is more common among the lower classes of society in the country than in London. It is not uncommon to see it pass through a village school, assuming a variety of characters, according to the state of the constitution of the child affected with it. I do not mean to say, that the pustule† is always imperfect, and not effective, when the inoculated patient has this malady; on the contrary, it is sometimes perfectly correct, and much more frequently so when it has been of

* The labors of Dr. Willan in this rugged field of science, all should acknowledge with thankfulness and gratitude.

† Having in my former Treatises used the term *pustule*, I make choice of it now, lest it should create confusion; though, perhaps, not so appropriate as *pock* or *vesicle*.

long standing, than when in its recent state; and what is remarkable, the disease is then (when of long duration) sometimes swept entirely away. I have noticed this impediment to the perfect formation and progress of the vaccine pustule in my general correspondence for more than two years past, and conceive it to be a more frequent source of the spurious pustule than any other, or indeed than all the rest united. Dr. Marcet inserted some hints I communicated to him on this head, in your Journal, for May, 1803, but I believe they have not been much attended to.

In my Paper of Instructions for Vaccine Inoculation, published some years back, I have endeavoured to guard the inoculator from being deceived by false appearances, by the following observations.

"The vaccine fluid is liable, from causes apparently trifling, to undergo a decomposition. In this state it sometimes produces what has been denominated the spurious pustule; that is, a pustule, or an appearance on the arm not possessing the characteristic marks of the genuine pustule. Anomalies assuming different forms may be excited, according to the qualities of the virus applied, or the state of the person inoculated; but by far the most frequent variety or deviation from the perfect pustule, is that which arrives at maturity, and finishes its progress much within the time limited by the true. Its commencement is marked by a troublesome itching; and it throws out a premature efflorescence, sometimes extensive but seldom circumscribed, or of so vivid a tint as that which surrounds the pustule completely organized; and (which is more characteristic of its degeneracy than the other symptoms) it appears more like a common festering produced by a thorn or any other small extraneous body sticking in the skin, than a pustule excited by the vaccine virus. It is generally of a straw colour, and when punctured, instead of the colourless, transparent fluid of the perfect pustule, its contents are found to be opaque. A little practice in vaccine inoculation attentively conducted, impresses on the mind the perfect character of the vaccine pustule; therefore, when a deviation arises, of whatever kind it may be, common prudence points out the necessity of re-inoculation." The deviation, when it arises from the cuticular disease I am speaking of, generally corresponds with that above recited. I might have added, that if the pustule is not much disturbed in its course by scratching, it commonly terminates in a scab of a pale brown or amber ber colour, and soft in its texture compared with that produced by the true vaccine pustule. I have abundant testimony to prove, that the fluid taken from a spurious vaccine pustule thus excited, is capable of propagating and perpetuating its like. Indeed, the vaccine fluid, even in a pustule going through its course perfectly, if taken in its far advanced stages, is capable of producing varieties, which will be permanent if we continue to vaccinate from it. I mention the subject briefly now; but it is my intention (as it embraces a wide field) to enlarge upon it, and some others connected with vaccination, when circumstances will permit me. Medical practitioners should be particularly circumspect when they inoculate those who have cuticular diseases. The danger of insecurity would be at once obviated, if on the appearance of an irregular pustule, the disease were to be subdued by proper applications, and the patient then reinoculated. I shall select a case, to show the efficacy of this mode of proceeding.

A family, consisting of five fine healthy-looking children, were inoculated by me at Cheltenham in the autumn of 1803 with fluid virus taken immediately from a proper vaccine pustule. On examining the punctures on the fifth day, I found, that on the left arm of one of the children, the pustule was advancing too rapidly. It was of an irregular form, contained already an opaque fluid, and was surrounded by an efflorescence of a dusky red colour to the extent of one-third part of an inch. Such an intolerable itching was excited, that the boy (who was only three years old) could not be prevented from rubbing it. This appearance led me to an examination, and on the child's head I observed an herpetic blotch not much larger in circumference than a shilling. The hair around the part was stiffened by the concreted ichor oozing from the sore, which had made its appearance about ten days. No eruption shewed itself in any other part of the body. The pustule was repeatedly broken by the child's scratching and rubbing it; and the inflammation on the arm, which began to spread so early, on the eighth and ninth day became very extensive. The child, at the same time, was hot and restless. A soft, amber-coloured scab* now began

* It may be remarked, that purulent matter cannot form a scab so hard and compact as limpid matter. Hence arises the difference between the variolous and the vaccine scab. It accounts too for the varicellous scab being commonly harder than the variolous.

gan to form; but this being rubbed off, the part ulcerated and healed slowly, leaving a cicatrix deeper and larger than in ordinary cases. The disease on the scalp was now quickly subdued by the use of tar ointment; and at the expiration of six weeks from its commencement, the inoculation was repeated, when the pustule went through all its proper stages with perfect regularity. The rest of the children inoculated at the same time, went through the cow-pox in the ordinary way, without any irregular appearance.

I have selected this case, to shew how slight a local appearance may produce a change in the state of the skin, at a distance from it. I cannot call it a general change in every case, as I have sometimes found a correct pustule on one arm, and a spurious pustule on the other; indeed, I have sometimes found the perfect and imperfect pustule on the same arm, within two inches of each other, when the virus inserted was taken the same instant from the same perfect pustule. It happens that I more frequently detect the disease by the appearance of the arm, than previously to inoculation. Parental fondness is often mismanaged, and induces mothers to conceal eruptive complaints on their children.

These are the constitutions which sometimes shew a few wandering pustulous eruptions after Vaccine Inoculation; and so peculiarly irritable is the skin when influenced by herpes, that the smallest wound, a slight scratch, or the pricking of a pin, for example, commonly produces inflammation, and slight, superficial suppuration.

The preceding year I inoculated another child at Cheltenham, whose face was involved in one general thick incrustation. She had been in this state, without any material variation, upwards of two years, during which time many applications had been used to no purpose. The scalp partook, in some degree, of the same kind of disease; but the body and limbs were free from it, except when any of the acrid fluid, oozing from fissures in the crust, chanced to fall upon the neck or breast; it then invariably produced, for a time, a similar appearance. On vaccinating this child by a single puncture in each arm, the pustules went through their course correctly. On their decline, the incrustation began to be less coherent, and to drop off; and at the expiration of a fortnight, the face was smooth, no vestige of the disease remaining, except a slight inflammation of the eye-lids.

Cases of this sort have become familiar; Mr. Ring has given several in his very copious Treatise on the Cow-pox; and they have been mentioned by other authors, both here and on the Continent.

I have in like manner sometimes seen papulous eruptions, which have long proved troublesome, speedily swept away.

This I think may be accounted for. The vaccine virus, a few days after its insertion, begins to act upon the constitution. It is then manifest, from a new appearance which these eruptions put on, commonly that of increased inflammation, that a new action has been excited in them. The original morbid action therefore becomes deranged and is destroyed, and consequently the disease is conquered. I have seen many instances where pre-existing pimples have been converted into vaccine pocks, which have kept pace with those on the arms in their progressive changes.

Seeing that the skin, when disposed to reject the ordinary action of the variolous virus, rejects the vaccine also, I shall just observe, it occurs to me as probable, that its herpetic state, at the time of inoculation, has been the chief source of those failures, which many practitioners have witnessed in inoculating for the small-pox: for in many instances where, on subsequent exposure to infection, the disease has been taken, it has been found that the process of inflammation and suppuration on the arms had gone to a greater extent than in ordinary cases, that the symptomatic affections were clearly marked, and that even eruptions, though small and seldom maturating, have appeared. But as the state of the arm became a secondary object in inoculating for the small-pox, our solicitude being directed to what appeared of far more consequence, the number of pustules, I almost despair of obtaining much information on this point.

I shall conclude this paper by observing, that although the Vaccine Inoculator does not inflict a severe disease, but, on the contrary, produces a mild affection scarcely meriting that term, yet, nevertheless, he should be extremely careful to obtain a just and clear conception of this important branch of medical science. He should not only be acquainted with the laws and agencies of the vaccine virus on the constitution, but with those of the variolous also, as they often interfere with each other.

A general knowledge of the subject is not sufficient to enable or to warrant a person to practice Vaccine Inoculation; tion; he should possess a particular knowledge; and that which I would wish strongly to inculcate, as the great foundation of the whole, is an intimate acquaintance with the character of the true and genuine vaccine pustule. The spurious pustule would then be readily detected, whatever form it might assume; and errors known no more.

I am, &c.

*Berkeley, July* 15, 1804. EDWARD JENNER.

